# Adaptive Local Aspect Dictionary Pair Learning for Synthetic Aperture Radar Target Image Classification

**DOI:** 10.3390/s18092940

**Published:** 2018-09-04

**Authors:** Xinzheng Zhang, Zhiying Tan, Guo Liu, Hongqing Liu, Yijian Wang, Shujun Liu, Yongming Li, Hao Xu, Jili Xia

**Affiliations:** 1 College of Communication Engineering, Chongqing University, Chongqing 400044, China; 20161202031t@cqu.edu.cn (Z.T.); 20114898@cqu.edu.cn (G.L.); 20161202029t@cqu.edu.cn (Y.W.); ly007@cqu.edu.cn (S.L.); yongmingli@cqu.edu.cn (Y.L.); 201712131083@cqu.edu.cn (J.X.); 2 Chongqing Key Lab of Mobile Communications Technology, Chongqing University of Posts and Telecommunications, Chongqing 400065, China; hongqingliu@outlook.com; 3 Spacecraft General Design Department, China Academy of Space Technology, Beijing 100094, China; haibeihms@163.com

**Keywords:** SAR, images classification, dictionary learning, representation learning, aspect

## Abstract

In this paper, a new target classification algorithm based on adaptive local aspect dictionary pair learning for synthetic aperture radar (SAR) images is developed. To that end, first, the aspect sector of one testing sample is determined adaptively by a regularized non-negative sparse learning method. Second, a synthesis dictionary and an analysis dictionary are jointly learned from the corresponding training subset located in the aspect sector. By doing so, the local aspect dictionary pair is obtained. Finally, the class label of the testing sample is inferred by a use of the minimum reconstruction residual under the representation with the local aspect dictionary pair. Using the local aspect sector training subset rather than the global aspect training set reduces the interference of a large amount of unrelated training samples, which leads to a more discriminative local aspect dictionary pair for target classification. The experiments are conducted with the Moving and Stationary Target Acquisition and Recognition (MSTAR) database, and the results demonstrate that the proposed approach is effective and superior to the state-of-the-art methods.

## 1. Introduction

Synthetic aperture radar (SAR) works regardless of light and weather conditions, and observes the Earth’s surface all day [1,2]. SAR is widely applied in various civil and military fields such as resource exploration, ecological environment monitoring, climate change research, military mapping, and military reconnaissance [3,4,5]. With the continuous developments of SAR technology, automatic target recognition (ATR) [6,7,8] of SAR images has attracted great attention over the years. The SAR images are attributed to electromagnetic scattering, which is not visual and hard to interpret directly, and they are also sensitive to aspect and depression angles [9]. This means few varieties in these angles cause significant changes in target images, which increases the difficulty of the classification.

In general, an integrated SAR ATR system may consist of three stages: detection, discrimination, and classification. Detection and discrimination will reject the clutter false alarms and will select out the image chips, that is, regions of interest (ROIs), containing candidate targets. The ROIs are sent to the classifier to decide the target class [10,11]. In the literature, the automatic SAR target recognition technology generally includes the traditional template matching method, the model-based algorithm, and the methods based on features such as principal component analysis (PCA) [12,13], wavelet transform [14,15], radon transform [16]. In addition, considering the scattering characteristics of SAR images, there are two typical models: conditionally Gaussian model [11] and scattering centers model [17]. The conditionally Gaussian model considers a stochastic signal model and forms a complex Gaussian random process under treating a SAR image as a column vector. The scattering center model provides a concise and physically relevant description of the target radar signature. For ground targets, a global scattering center model is proposed in [18], which is established offline using range profiles at multiple viewing angles. In this paper, we focus on the model based machine learning and tread the SAR images as a two-dimensional data matrix.

With the developments of machine learning, it has been successfully applied in SAR target images classification as well. Recently, sparse representation becomes a useful technique to represent signals by a linear combination of a series of known signals, where the representation coefficients are sparse [19,20]. The classification model based on sparse representation provides advantages of high recognition rate and robustness to strong noise. Particularly, Zhang et al. [21] proposed a multi-view joint sparse representation method for SAR ATR. The advantages of this method are exploiting the correlation among multiple views of the same target in different aspects without knowing the pose and achieving better recognition results. Dong et al. [22,23] studied the SAR target recognition method based on joint sparse representation with monogenic features, and then developed the approach on the Grassman manifold, which exploits similarity between the sets of monogenic components on Grassmann manifolds for target recognition and avoids high dimension and redundancy. Liu et al. [24] investigated the Dempster-Shafer fusion of multiple sparse representations for SAR target images recognition, which can describe both the detail and global features of targets. This method makes use of the prior information and dictionaries are constructed by using the samples of each configuration to better capture the detail information of the SAR images. Although sparse representation has obtained promising results, there still are some inevitable problems in practice. For example, dictionaries of sparse representation are usually composed of global aspect training samples, leading to high storage and calculation costs. To solve this issue, the dictionary learning technique can be utilized. In a natural image recognition application, Jiang et al. [25] proposed a label consistent K-singular value decomposition (LC-KSVD) algorithm and introduced a binary class label sparse code matrix to challenge samples from the same class with similar sparse codes, which obtained the optimal solution efficiently in experiments. In 2014, Gu et al. [26] developed a projective dictionary pair learning (DPL) framework to learn a synthesis dictionary and an analysis dictionary jointly to achieve the goal of signal representation and discrimination, and dictionary pair learning process avoids *l*_0_-norm or *l*_1_-norm optimization, and reduces the time complexity in the training and testing phase. Projection transform method is very effective in computer vision. Kahaki [27] proposed mean projection transform (MPT) as a corner classifier, which presented fewer false-positive (FP) and false-negative (FN) points. What’s more, the output results of the corner classifier exhibit better repeatability, localization, and accuracy of repeatability for the detected points compared with the criteria in original and transformed images. For SAR target image recognition, Sun et al. [28] proposed a SAR image target recognition method based on dynamic sparse representation and dictionary learning. The learned dictionaries have smaller sizes and are more distinctive among different classes, which can speed up recognition and improve the accuracies. Song et al. [29] reported a sparse representation SAR target recognition algorithm with the supervised discrimination dictionary learning based on histogram of oriented gradients (HOG) features. The method can reliably capture the structures of targets in SAR images and achieves a state-of-the-art performance. Liu et al. [30] introduced a new scattering center feature extraction and target recognition methods based on sparse representation and dictionary refinement to decrease the cost of computation and storage. It is of interest to note that, in these above SAR target recognition approaches based on dictionary learning, global aspects training samples are all used as atoms, by doing which aspect characteristics of SAR target are neglected. Of cause, there are still other methods using different local information. For instance, Liu et al. [31] proposed a novel method based on deep belief network and local spatial information for polarimetric SAR (POL-SAR) image classification, which makes full use of the prior knowledge of POL-SAR data and overcomes shortcomings of traditional methods sensitive to extracted features and slow to execute. Cao et al. [32] developed a method of joint sparse representation of heterogeneous multi-view SAR images over a locally adaptive dictionary, in which high recognition accuracy is guaranteed by combination of more target information and adjustment of the inter correlation information guarantee.

**Figure 1:**
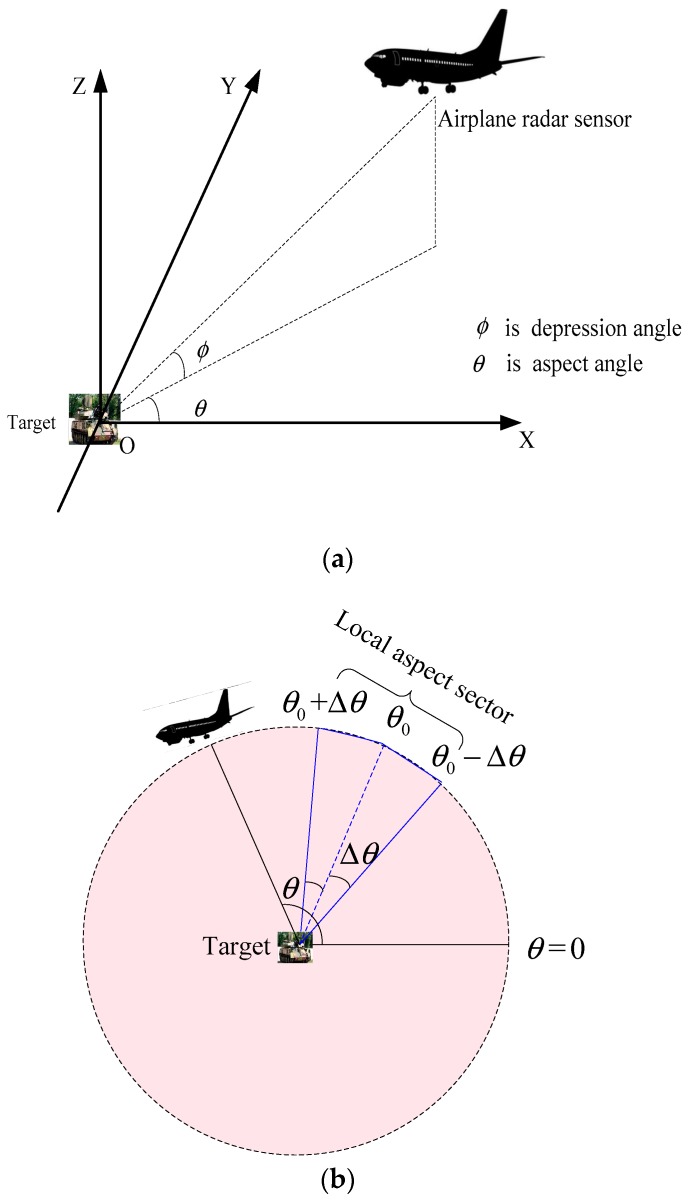

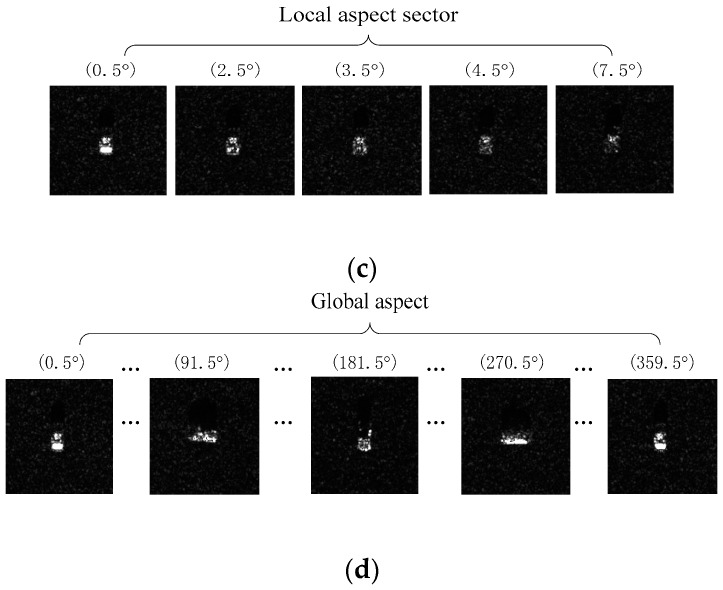
(**a**) Description of aspect angle and depression angle in radar sensor. (**b**) Top view of aspect. (**c**) SAR target images in local aspect sector. (**d**) SAR target images in global aspect.

In this paper, the intention is to exploit the local aspect characteristics of SAR targets. Based on the projective dictionary pair learning algorithm, a new approach for SAR target images classification is proposed. Figure 1 gives a brief depiction about the concept of local aspect. Figure 1a describes the aspect angle and depression angle when radar sensors imaging. Figure 1b visually describes the local aspect sector and global aspect. The local aspect means a small range of aspects variation, in which target scattering characteristics does not change significantly leading to similar images as shown in Figure 1c. On the contrary, SAR images with large aspect difference from 0.5° to 359.5° are distinct each other as shown in Figure 1d. As discussed early, in the most of the previous SAR target images recognition methods, images acquired at all aspect angles from 0° to 360° for the same target are considered to be equally correlated. In other words, there is no consideration on the likelihood difference of various aspects training samples and the test sample.

The aforementioned sparse representation-based recognition models assume that a test sample is represented by a linear representation of global aspect training samples. However, this assumption is not really reasonable. In fact, for the same target, when its relative position with the radar varies, the changes of its scattering structure lead to the changes of the strong scattering point position and scattering intensity, which generates the changed echoes. When the aspect changes greatly, the echoes of the target are obviously different. Therefore, the SAR target images are closely related to the aspects when target is being imaged, and the information of two images in different aspects is very different. According to the characteristics of SAR target images, global aspects training samples are actually in a nonlinear manifold. However, because the structural scattering of the target is stable over a small range of local aspects, the test sample can be represented linearly by the training samples near the corresponding local aspect sector.

**Figure 2:**
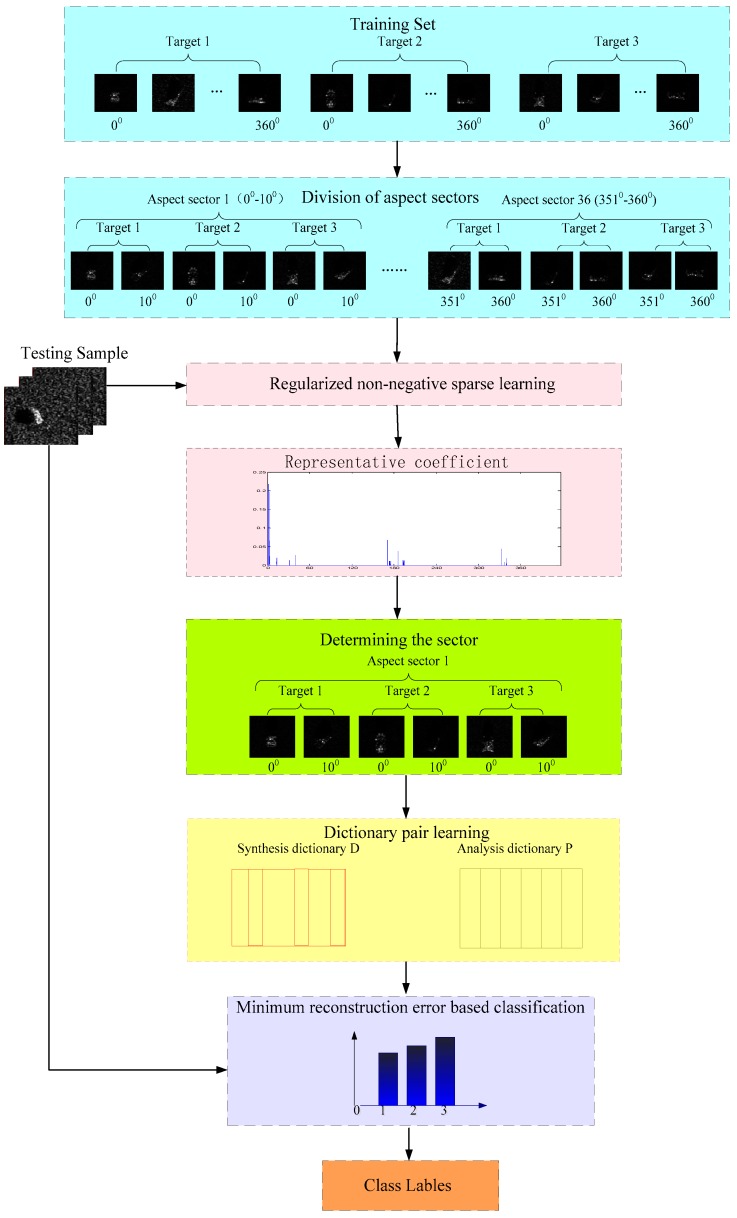
The scheme of adaptive local aspect dictionary pair learning based SAR target images classification.

The main contribution of this paper is to propose a new SAR target images recognition method based on adaptive local aspect dictionary pair learning. Figure 2 shows a scheme of the proposed method. As seen in Figure 2, first, the global aspect range is divided into multiple local aspect sectors. For the current testing sample, the local aspect sector is adaptively determined based on regularized non-negative sparse learning according to its representation coefficient in the middle graph. Then, a dictionary pair including a synthesis dictionary and an analysis dictionary is learned from the training subset in the local aspect sector. Finally, under the local discriminate dictionary pair obtained from the training phase, the class label of the test sample is determined with the minimum reconstruction error. The mechanism behind this method is that the training subset in the local aspect sector satisfies the local linear representation qualification. The learned dictionary pair has better interclass discrimination ability. In addition, the interference of training samples outside the local aspect sector is excluded, which further improves the recognition performance. The experiments based on Moving and Stationary Target Acquisition and Recognition (MSTAR) dataset are conducted and the results verify the effectiveness and superiority of the proposed method.

This paper is organized as follows: in Section 2, the selection of adaptive local aspect sector based on regularized non-negative sparse learning is introduced. In Section 3, the dictionary pair learning and recognition method based on adaptive local aspect is proposed. The experimental results and analysis of the proposed method are provided in Section 4. The conclusions are drawn in Section 5.

## 2. Adaptive Local Aspect Sector Selection

As mentioned in the introduction, the global aspect training samples of SAR target actually lie in a non-linear manifold space. For a test sample, if its aspect angle is *θ*_0_, only those training samples in the local aspect sector nearby *θ*_0_ can linearly represent the current test sample. The key issue is to find the correct local aspect sector of the current test sample. In this paper, we propose a regularized non-negative sparse learning approach to solve this problem. Assuming that the aspect of the current test sample ***y***_0_ is *θ*_0_, it can be represented linearly by $n_{\theta_{0}}$ training samples $x_{1,\theta_{0}},\;x_{2,\theta_{0}},...,x_{n_{\theta_{0}},\theta_{0}}$ located in local aspect sector $\left(\theta_{0}-\Delta \theta ,\theta_{0}+\Delta \theta \right]$. The representation is:

(1) $$y_{0}=x_{1,\theta_{0}}\alpha_{1,\theta_{0}}+x_{2,\theta_{0}}\alpha_{2,\theta_{0}}+...+x_{n_{\theta_{0}},\theta_{0}}\alpha_{n_{\theta_{0}},\theta_{0}}$$

where $\alpha_{\theta_{0}}={\left[\alpha_{1,\theta_{0}},\alpha_{2,\theta_{0}},\ldots ,\alpha_{n_{\theta_{0}},\theta_{0}}\right]}^{T}$ is the coefficient vector of $y_{0}$ over $x_{1,\theta_{0}},\;x_{2,\theta_{0}},...,x_{n_{\theta_{0}},\theta_{0}}$. Since the true aspect of the test sample is unknown, it should be represented by all aspect training samples from 0°–360°. To obtain the sparsest solution, we can turn the problem into an optimization problem with the *l*_0_-norm constraint, given by:

(2) $$\begin{array}{l} \underset{\alpha }{\min}{\left\|\alpha \right\|}_{0}\\ s.t.\;{\left\|y_{0}-X\alpha \right\|}_{2}^{2}\leq \varepsilon \end{array}$$

where X denotes the entire training set, α represents the coefficient vector, and ε indicates the tolerance. ${\left\|\cdot \right\|}_{0}$ represents the *l*_0_-norm, and ${\left\|\cdot \right\|}_{2}$ denotes the *l*_2_-norm.

**Figure 3:**
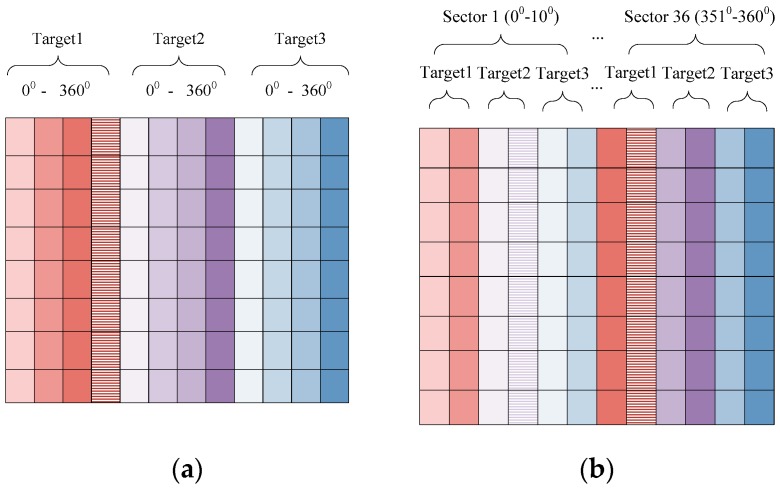
Dictionary structure. (**a**) Traditional dictionary. (**b**) Proposed dictionary with local aspect sectors.

According to the sparse learning theory, if we sparsely regularize and constrain with all aspect sectors training samples, the elements in the coefficient vector corresponding to atoms with the same aspect sector as the test sample should be non-zero values, while other coefficient elements are zeros [19,20]. To effectively represent the training samples, a new dictionary is constructed based on the aspect angles instead of the traditional class labels. In traditional dictionaries, the order of atoms is arranged according to their class labels, and the order of the same class has no relevance with the aspect angle and is arranged randomly. For instance, the aspect sector interval is set to 10°. In this work, the dictionary atoms are arranged according to aspect angles as Target1 (0°–10°); Target2 (0°–10°); Target3 (0°–10°); Target1 (11°–20°); Target2 (11°–20°); Target3 (11°–20°); …; Target1 (351°–360°); Target2 (351°–360°); Target3 (351°–360°). The difference between the traditional dictionary construction and the dictionary constructed with local aspect sectors are displayed in Figure 3.

Moreover, in order to comply with the physical meaning of representation learning, the non-negative constraints to the representation coefficient vector are added. Since the optimization of *l*_0_-norm minimum problem is NP-hard, the problem is usually solved by minimizing the *l*_1_-norm. Moreover, to make the elements of the coefficient vector more likely to be in a probabilistic sense, we introduce the constraint of the sum of all elements in each representation coefficient, that is $\sum_{i=1}^{n}\alpha_{i}=1$. With this constraint, the value of each elements in representation coefficient can evaluate the contribution of each training sample for representing the $y_{0}$. Therefore, the final model for adaptively selecting local aspect sectors based on regularized non-negative sparse learning is:

(3) $$\begin{array}{l} \underset{\alpha^{A}}{\min}{\left\|X^{A}\alpha^{A}-y_{0}\right\|}_{2}^{2}+\lambda {\left\|\alpha^{A}\right\|}_{1}\\ s.t.\;\alpha_{i}\geq 0,\sum_{i=1}^{n}\alpha_{i}=1 \end{array}$$

where $X^{A}$ donates the dictionary designed according to aspect angles, $\alpha^{A}={\left[\alpha_{1},\alpha_{2},\ldots \alpha_{i},\ldots ,\alpha_{n}\right]}^{T}$ is the coefficient vector. n is the total number of training sets. ${\left\|\cdot \right\|}_{1}$ denotes the $\ell_{1}$-norm.

In this way, for each test sample, after obtaining the coefficient vector, the sum of coefficient elements for each local aspect sector can be calculated, which can be seen as the efforts corresponding to each local aspect sector. According to the aforementioned analysis, it is reasonable to infer that the current test sample corresponds to the local sector $s_{y}$ with the maximum sum, which indicates that the aspect sector corresponding to a test sample can be determined adaptively. The formula is written as follows:

(4) $$s_{y}=\underset{s}{\max}\sum_{1}^{n_{s}}\alpha_{i}$$

where $n_{s}$ is the number of samples of each sector.

To efficiently obtain the solution, the accelerated projected gradient method [33] is employed to optimize the model (3). It should be noted that the purpose of the regularized non-negative sparse learning is not to obtain the class label of the test sample, but to adaptively choose the local aspect sectors where the test sample is located.

**Figure 4:**
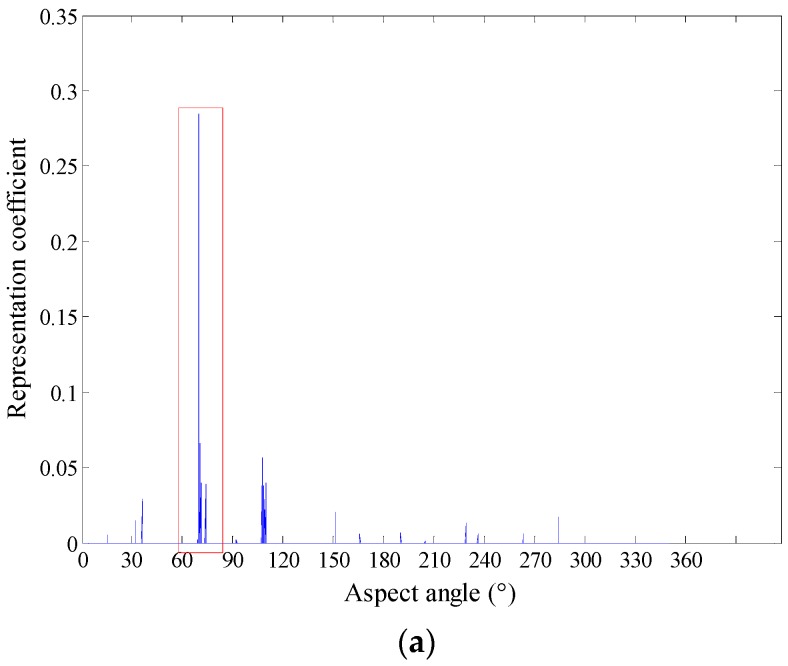

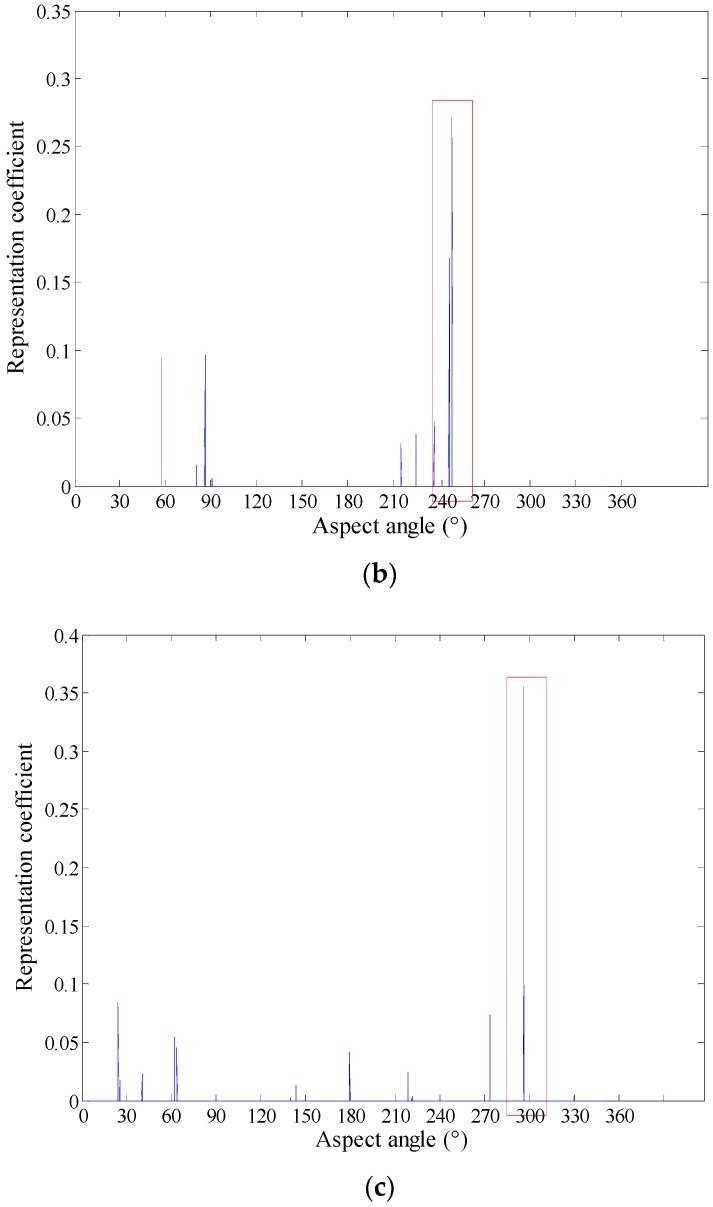
(**a**) The representation coefficient of Target1 (68.5°). (**b**) The representation coefficient of Target2 (240.0°). (**c**) The representation coefficient of Target3 (293.8°).

To clearly illustrate our idea, the following experiment is conducted to show the local aspect sector selection process. For a test sample of Target1, its real aspect angle is 68.5°. Because we divide the global aspect to local aspect sector by 10°, the sample should be located in the (60°, 70°) sector. The coefficient vector obtained by the regularized non-negative sparse learning method is shown in Figure 4a. It is seen that the elements corresponding to the (60°, 70°) sector is obviously larger than others, which indicates the local aspect sector of the test sample is correctly determined. Figure 4b,c show the experimental results of a Target2 test sample (240.0°) and Target3 test sample (293.8°) respectively. As shown in these figures, although the test samples are from different targets, the local aspect sector of them can be inferred exactly and adaptively.

## 3. DPL Based on Local Aspect Sector

### 3.1. DPL

Dictionary learning simplifies learning task and reduces the model complexity through an appropriate dictionary, which has been widely studied in various pattern classification problems. Because of its efficiency, the projective dictionary pair learning method [26] is utilized to classify SAR image targets.

Assume that the training set of *p*-dimension from the K class, S sectors is denoted by $X^{A}=[X^{1},...,X^{s},...,X^{S}]$, $X^{s}\in {ℜ}^{p\times n_{s}}$ is the training samples set of the *s*-th sector. The traditional discrimination dictionary learning method [26] is expressed as:

(5) $$\min_{D,A}{\left\|X^{A}-DA\right\|}_{F}^{2}+\lambda {\left\|A\right\|}_{p}+\Phi \left(D,A,L\right)$$

where λ≥0 is a scalar constant, L represents the class label matrix of the sample in $X^{A}$, D is the synthesis dictionary, and A is the matrix of coding coefficients of $X^{A}$ over D. In the model (5), the constraint term ${\left\|X^{A}-DA\right\|}_{F}^{2}$ ensures the representation ability of D, ${\left\|A\right\|}_{p}$ is the *l_p_*-norm, Φ(D,A,L) represents the discriminative promotion function and ensures the discriminative ability of D and A.

However, the above model still employs *l*_1_-norm sparse regularization on the coding coefficients. To avoid the *l*_1_-norm sparse solution process, in this work, an analysis dictionary $P\in {ℜ}^{mK\times p}$, is employed so that the code matrix A can be analytically obtained as $A=PX^{A}$. In such a way, synthesis dictionary D and analysis dictionary P are jointly learned, given by:

(6) $$\left\{P^{*},D^{*}\right\}=\underset{P,D}{\arg\;\min}{\left\|X^{A}-DPX^{A}\right\|}_{F}^{2}+\Phi \left(D,P,X^{A},L\right)$$

where $\Phi \left(D,P,X^{A},L\right)$ donates a discriminant function, D and P form a dictionary pair: the analysis dictionary P is used to analytically code $X^{A}$, and the synthesis dictionary D is used to reconstruct $X^{A}$.

The discriminating power of the DPL model depends on the rational design of $\Phi \left(D,P,X^{A},L\right)$. One rational consideration is that since the representation is structured, synthesis dictionary and analysis dictionary should be structured as well, namely they should present the forms of $D=[D_{1},...,D_{k},...,D_{K}]$ and $P=[P_{1},...,P_{k},...,P_{K}]$, where $\left\{D_{k}\in {ℜ}^{p\times m},P_{k}\in {ℜ}^{m\times p}\right\}$ is a sub-dictionary pair corresponding to the *k*-th class.

In the proposed DPL, the projective dictionary $P_{k}$ is designed such that samples corresponding to the same class *k* can be well represented. That is, the energy of $P_{k}X_{k}^{A}$ will be much larger than $P_{k}X_{i}^{A}$, ∀k≠i. Obviously, under this condition, the matrix PX will approximate a block diagonal matrix. In the same thinking, $D_{k}$ will be able to reconstruct the data matrix $X_{k}^{A}$ from the projection coding matrix $P_{k}X_{k}^{A}$. Based on these considerations, the synthesis dictionary and analysis dictionary are designed as:

(7) $$\underset{P,D}{\min}\sum_{k=1}^{K}{\left\|X_{k}^{A}-D_{k}P_{k}X_{k}^{A}\right\|}_{F}^{2}$$

With the designed synthesis dictionary and analysis dictionary, the DPL model finally is

(8) $$\left\{P^{*},D^{*}\right\}=\arg\;\underset{P,D}{\min}\sum_{k=1}^{K}{\left\|X_{k}^{A}-D_{k}P_{k}X_{k}^{A}\right\|}_{F}^{2}+\lambda {\left\|P_{k}{\bar{X}}_{k}^{A}\right\|}_{F}^{2},s.t.{\left\|d_{i}\right\|}_{2}^{2}\leq 1$$

where ${\bar{X}}_{k}^{A}$ represents the sample except the entire training set $X_{k}^{A}$ and $d_{i}$ is the *i*-th atom of the synthesis dictionary D. To avoid the solution of $P_{k}=0$, the energy constraint of each atom $d_{i}$ in (8) is added.

### 3.2. DPL Projective Dictionary Pair Learning Based on Local Aspect Sector

As discussed early, the dictionary pair utilized to recognize the current test sample is learned from the training subset in the corresponding local aspect sector. Therefore, from the sector point of view, the objective function of DPL model is:

(9) $$\left\{P^{s},D^{s}\right\}=\arg\;\underset{P^{s},D^{s}}{\min}\sum_{k=1}^{K}\left[{\left\|X_{k}^{s}-D_{k}^{s}P_{k}^{s}X_{k}^{s}\right\|}_{F}^{2}+\lambda {\left\|P_{k}^{s}{\bar{X}}_{k}^{s}\right\|}_{F}^{2}\right],s.t.\;{\left\|d_{k,n_{i}}^{s}\right\|}_{2}^{2}\leq 1$$

where the superscript s indicates the corresponding *s*-th sector. The whole process of learning a dictionary pair is to solve this objective function.

According to aspect intervals, we divide the global aspect training set into *s* aspect sections. This provides one an opportunity to explore the training sample local aspect scattering characteristics. By a use of the aspect information of test samples, the aspect correlation within the same class is enhanced and differences across classes are also increased.

In (9), the given optimization is non-convex and to efficiently solve the problem, an auxiliary variable matrix $A^{s}$ is introduced to relax the optimization in (9). That is:

(10) $$\begin{array}{c} \left\{P^{s*},D^{s*},A^{s*}\right\}=\arg\;\underset{P^{s},D^{s},A^{s}}{\min}\sum_{k=1}^{K}\left[{\left\|X_{k}^{s}-D_{k}^{s}P_{k}^{s}X_{k}^{s}\right\|}_{F}^{2}+\tau {\left\|P_{k}^{s}X_{k}^{s}-A_{k}^{s}\right\|}_{F}^{2}+\lambda {\left\|P_{k}^{s}{\bar{X}}_{k}^{s}\right\|}_{F}^{2}\right]\\ s.t.\;{\left\|d_{k,n_{i}}^{s}\right\|}_{2}^{2}\leq 1 \end{array}$$

where τ is a scalar constant. For the optimization problem in (10), we can now alternatively update $A^{s}$ and $\left\{D^{s},P^{s}\right\}$ and the detailed steps are provided as follows.

For $A^{s}$, the object function can be rewritten as:

(11) $$A^{s*}=\arg\;\underset{A^{s}}{\min}\sum_{k=1}^{K}\left({\left\|X_{k}^{s}-D_{k}^{s}A_{k}^{s}\right\|}_{F}^{2}+\tau {\left\|P_{k}^{s}X_{k}^{s}-A_{k}^{s}\right\|}_{F}^{2}\right)$$

It is a simple quadratic programming and the closed-form solution is:

(12) $$A_{k}^{s*}={\left(D_{k}^{sT}D_{k}^{s}+\tau I\right)}^{-1}\left(\tau P_{k}^{s}X_{k}^{s}+D_{k}^{sT}X_{k}^{s}\right)$$

where I is the identity matrix.

For $\left\{D^{s},P^{s}\right\}$, one obtains:

(13) $$\left\{ \begin{array}{l} P^{s*}=\arg\;\underset{P^{s}}{\min}\sum_{k=1}^{K}\left(\tau {\left\|P_{k}^{s}X_{k}^{s}-A_{k}^{s}\right\|}_{F}^{2}+\lambda {\left\|P_{k}^{s}{\bar{X}}_{k}^{s}\right\|}_{F}^{2}\right)\\ D^{s*}=\arg\;\underset{D^{s}}{\min}\sum_{k=1}^{K}{\left\|X_{k}^{s}-D_{k}^{s}A_{k}^{s}\right\|}_{F}^{2},\;s.t.{\left\|d_{k,n_{i}}^{s}\right\|}_{2}^{2}\leq 1 \end{array}\right.$$

The closed-form solution of $P^{s}$ is:

(14) $$P^{s*}=\tau A_{k}^{s}X_{k}^{sT}{\left(\tau X_{k}^{s}X_{k}^{sT}+\lambda {\bar{X}}_{k}^{s}{\bar{X}}_{k}^{sT}+\mu I\right)}^{-1}$$

where μ is a small number. To optimize $D^{s}$ a variable M is introduced:

(15) $$\underset{M,D^{s}}{\min}\sum_{k=1}^{K}{\left\|X_{k}^{s}-D_{k}^{s}A_{k}^{s}\right\|}_{F}^{2},\;s.t.\;D=M,\;{\left\|m_{i}\right\|}_{2}^{2}\leq 1$$

To efficiently obtain the solution of (15), the alternating direction method of multipliers (ADMM) algorithm is utilized and the update steps are:

(16) $$\left\{ \begin{array}{l} D^{s\left(j+1\right)}=\arg\;\underset{D^{s}}{\min}\sum_{k=1}^{K}\left({\left\|X_{k}^{s}-D_{k}^{s}A_{k}^{s}\right\|}_{F}^{2}+\rho {\left\|D_{k}^{s}-M_{k}^{s}\right\|}_{F}^{2}\right)\\ M^{s\left(j+1\right)}=\arg\;\underset{M^{s}}{\min}\sum_{k=1}^{K}\rho {\left\|D_{k}^{s(j+1)}-M_{k}^{s}+G_{k}^{s(j)}\right\|}_{F}^{2},\;s.t.\;{\left\|m_{i}\right\|}_{2}^{2}\leq 1\\ G^{s\left(j+1\right)}=G_{k}^{s(j)}+D_{k}^{s(j+1)}-M_{k}^{s(j+1)},\;\mathrm{update}\;\rho \;\mathrm{if}\;\mathrm{appropriate}. \end{array}\right.$$

After solving the corresponding optimizations, the final label of the testing sample is determined using the minimum reconstruction error criterion by:

(17) $$l_{y}=\underset{k}{\min}{\left\|y-D_{k}^{s}P_{k}^{s}y\right\|}_{2}$$

**Figure 5:**
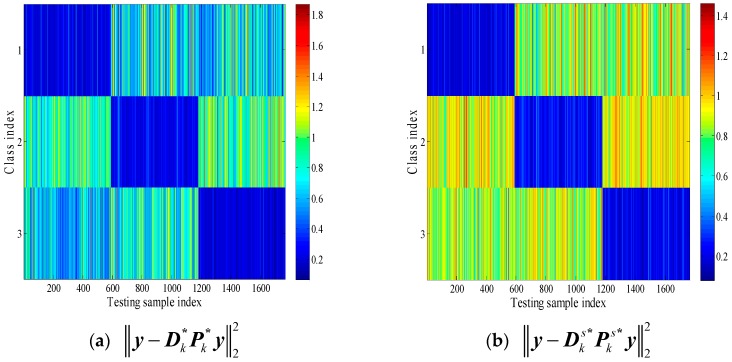
(**a**) Reconstruction error with global aspect training set. (**b**) Reconstruction error with local aspect sector training subset.

In Figure 5, the reconstruction residuals with global aspect training set and local aspect sector training subset are provided. It is seen that it maintains a typical block diagonal structure. Another observation is that the reconstruction residuals have great difference between classes, where with the local aspect sector training set, it demonstrates the inter-class diversities more effectively.

It is of interest to point out that in practical experiments, we can first learn the discrimination projective dictionary pairs of each local aspect sector to improve the efficiency of the algorithm. The steps of the proposed algorithm are now summarized in Algorithm 1.
**Algorithm 1:** Adaptive local aspect dictionary pair learning based SAR image classificationInput:X: All types of training samplesy: test samplesOutput: the identity of y
Steps: (1)Divide $X^{A}$ into $X^{s}$ according to the aspect sector intervals.(2)Calculate the representation coefficient $\alpha^{A}$ by (3).(3)Determine corresponding sector $s_{y}$ by (4).(4)Learn discrimination dictionary pair $D^{s}$, $P^{s}$ from training sample subset $X^{s}$ by (10).(5)Obtain the final label $l_{y}$ by (17).

### 3.3. Complexity and Convergence Analysis

The adaptive local aspect dictionary pair learning method (ALADPL) selects a smaller range of aspect domain as a subset, and reduces the interference caused by the atoms located in other aspect sectors compared to global aspect dictionary pair learning method (GADPL). In the stage of determining adaptive local aspect sectors, the sparse coefficient vector will converge in quadratic order in each iteration due to utilizing Newton acceleration, and the algorithm usually converges after a limited number of iterations. In the experiments, we found that the algorithm converged within 500 iterations. During learning dictionary pair stage, $A_{k}^{s}$, $P_{k}^{s}$ and $D_{k}^{s}$ are updated alternately. In each iteration, the time complexities of updating $A_{k}^{s}$, $P_{k}^{s}$ and $D_{k}^{s}$ are $O\left(mpn+m^{3}+m^{2}n\right)$, $O\left(mpn+p^{3}+mp^{2}\right)$ and $O\left(w\left(pmn+m^{3}+m^{2}p+p^{2}m\right)\right)$, respectively, where *w* is the iteration number in ADMM algorithm for updating $D^{s}$. In what follows, a series of experiments are conduced to verify the superiority of our proposed method compared with some state-of-the-art algorithms.

## 4. Experimental Results and Analysis

### 4.1. Introduction to Experimental Data Sets

**Figure 6:**
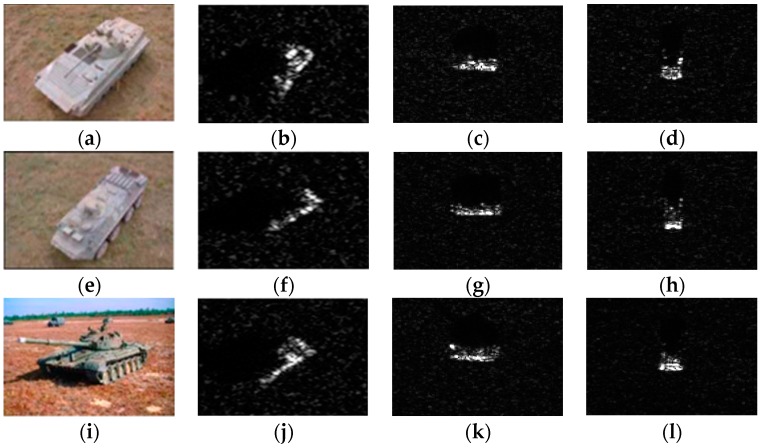
(**a**) Optical image of BMP2. (**b**) SAR image of BMP2 (45.5°). (**c**) SAR image of BMP2 (90.5°). (**d**) SAR image of BMP2 (181.5°). (**e**) Optical image of BTR70. (**f**) SAR image of BTR70 (45.0°). (**g**) SAR image of BTR70 (90.0°). (**h**) SAR image of BTR70 (180.0°). (**i**) Optical image of T72. (**j**) SAR image of T72 (44.8°). (**k**) SAR image of T72 (91.8°). (**l**) SAR image of T72 (180.8°).

The experimental data used in this work are the measured ground surface stationary target data released by the MSTAR program and supported by the Defense Advanced Research Projects Agency (DARPA) [34,35,36]. MSTAR SAR datasets are benchmark data for SAR image target classification algorithms evaluation. This dataset is collected by a high-resolution spotlight synthetic aperture radar with an imaging resolution of 0.3 m × 0.3 m and it operates in the X-band and adopts the HH polarization [37]. The collected data are preprocessed and are composed of many image chips, where each chip is a pixel size of 128 × 128 containing one target. SAR images in the database have an aspect range from 0° to 360°, and have two different depression angles (15°, 17°). Images acquired at 17° depression angle were used as training set, while images obtained at 15° depression angle used as test samples in this work. The experiment uses three major categories: BTR70 (armored transport vehicle), BMP2 (infantry fighting vehicle), and T72 (tank). Figure 6 shows the optical images of three targets and their SAR images at different aspect angles. BMP2 and T72 have several configurations [24], which have some differences in their deployments. For example, armored transport vehicles of the same class are different in barrels, fenders, and spotlights, while tanks of the same class are different in machine guns, fuel tanks, and antenna deployment and so on. To be specific, BMP2 has three configurations of BMP2-9563, BMP2-9566, and BMP2-C21, while BTR70 has one configuration of BTR70-C71, and T72 has three configurations of T72-132, T72-812 and T72-S7. The detailed types and numbers of training and test samples are shown in Table 1.

In the experiments, in order to reduce clutter interference around the target, a 52 × 52 sub-image centered at each original chip was extracted. In practical application, it is necessary to face classifying multiple targets, including structures, weapons, military vehicle and so on. Our research mainly focuses on military vehicle classification, but the proposed method could be adopted to classifying other structures or weapons. In addition, if the images contain two or three targets, we need to separate these targets first and then identify them separately.

### 4.2. Performance Comparison between ALADPL and GADPL

**Figure 7:**
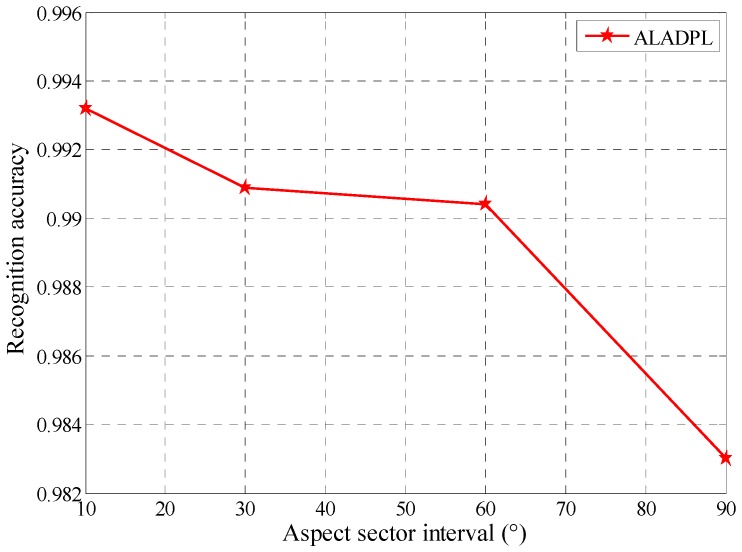
The recognition accuracy of ALADPL with different local aspect sector interval.

From the aforementioned analysis, we determine the local aspect sector of a test sample by regularized non-negative sparse learning firstly. Then, we learn the dictionary pair from the determined aspect sector according to Equation (9) and the dictionary learning process is to solve the objective function by Equations (10)–(16). So the division of aspect sectors in ALADPL has an important influence on classification performance. Generally speaking, the closer the azimuth angle is, the higher the correlation of SAR target images is. Dividing sectors according to different intervals produces different training subsets, and then generates different dictionary pairs. In the experiments, we divide the entire azimuth (0°, 360°) with different intervals including 10°, 30°, 60°, and 90° to demonstrate the performance of the ALADPL. Other regularization parameters are set as follow: τ set to 0.01, λ set to 0.01, μ  set to 0.0001. The result is depicted in the Figure 7. GADPL does not utilize the aspect information and its classification accuracy is 96.66%. For the proposed ALADPL, its accuracy drops as the interval increases. When the interval decreases, the recognition rate increases from 98.30 to 99.32%. The classification accuracy of the ALADPL is consistently higher than that of the GADPL, which indicates the learned dictionary pair reflects local aspect scattering characteristics of the target effectively.

**Figure 8:**
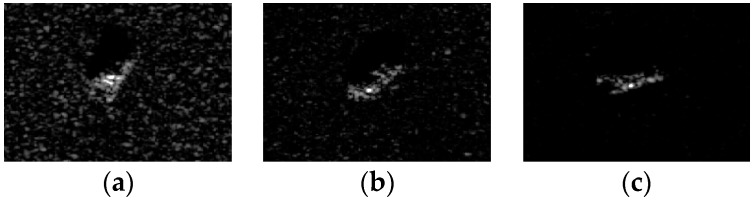
(**a**) BMP2 original SAR image. (**b**) BTR70 original SAR image. (**c**) T72 original SAR image.

**Figure 9:**
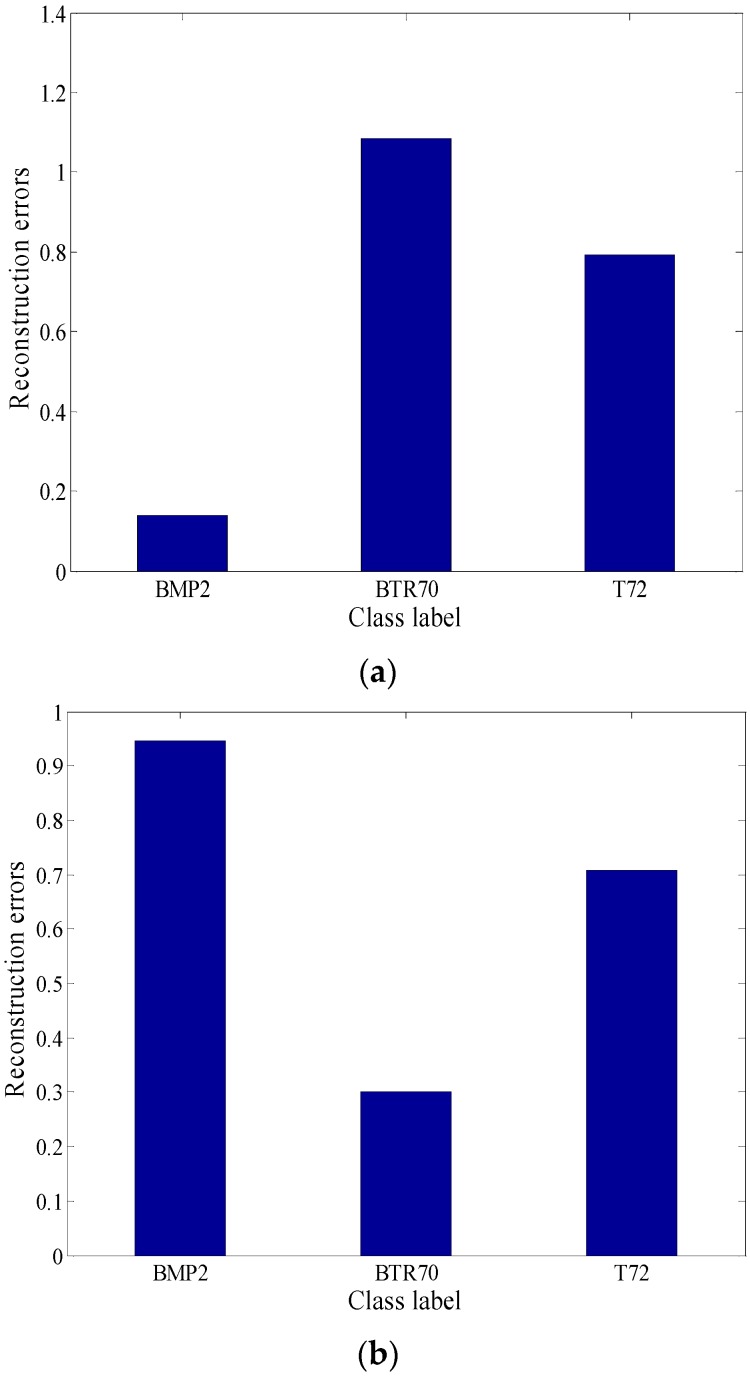

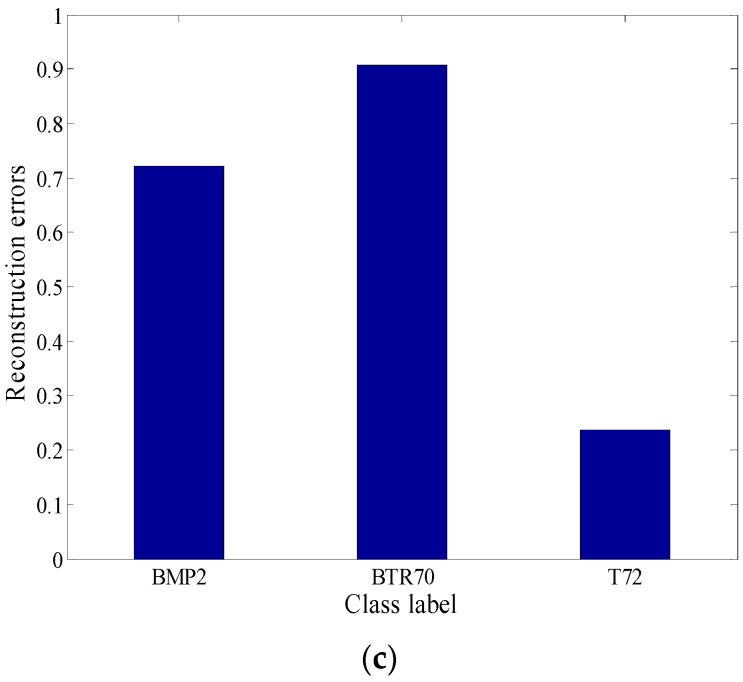
(**a**) BMP2 reconstruction errors of ALADPL. (**b**) BTR70 reconstruction errors of ALADPL. (**c**) T72 reconstruction errors of ALADPL.

**Figure 10:**
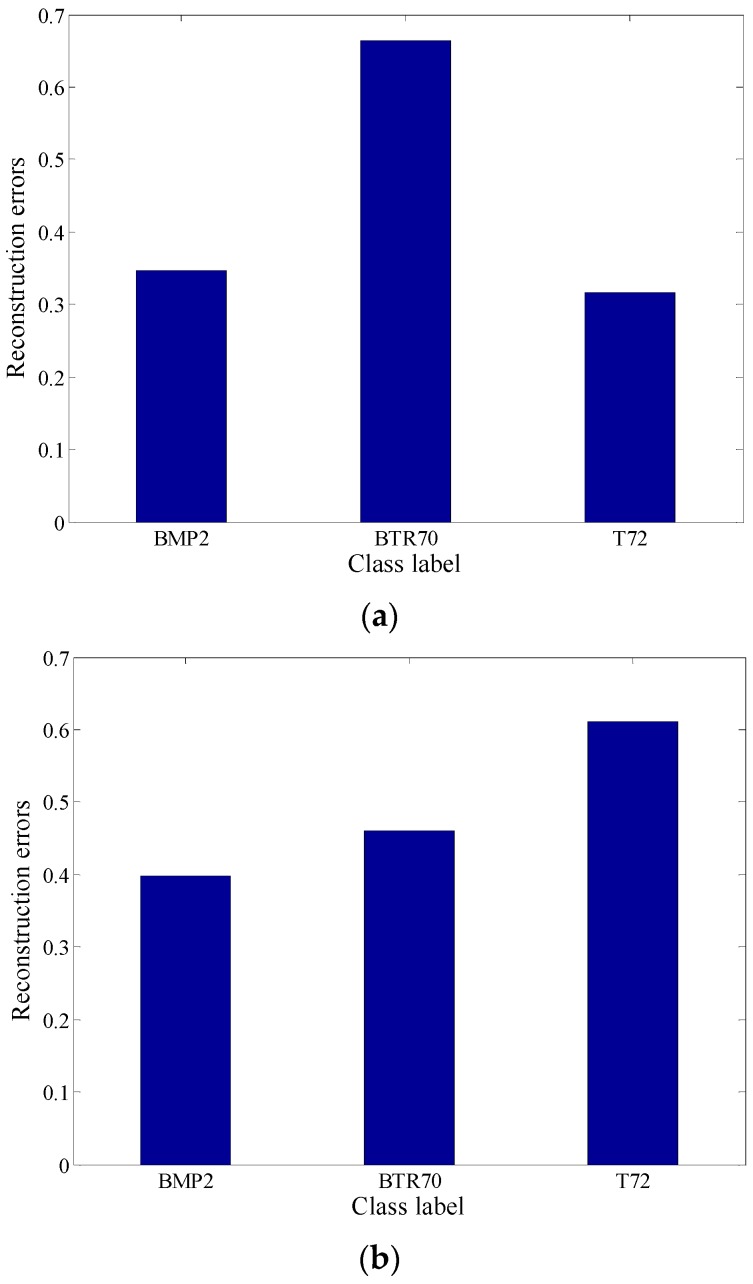

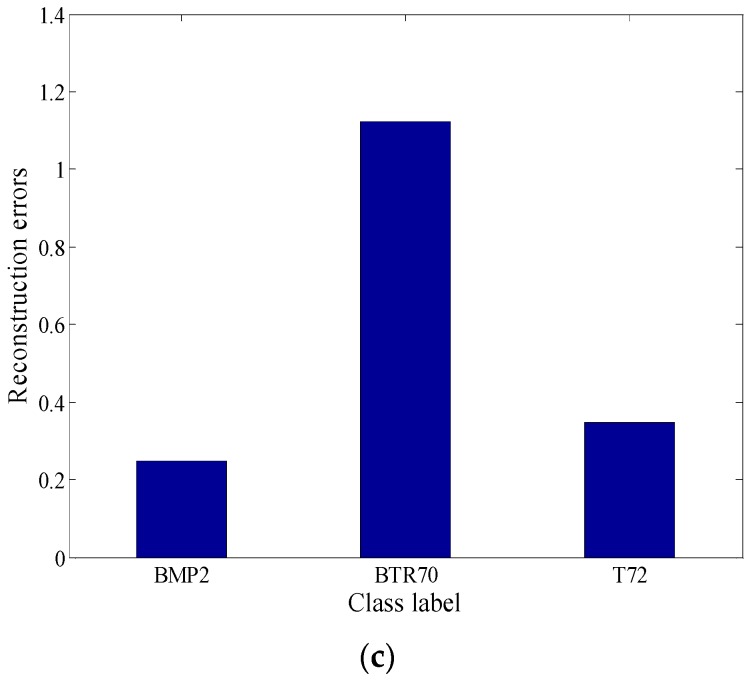
(**a**) BMP2 reconstruction errors of GADPL. (**b**) BTR70 reconstruction error of GADPL. (**c**) T72 reconstruction error of GADPL.

Further, several reconstruction error figures of three test samples are given as follows. In Figure 8, SAR images of these three test samples are shown from three targets separately. Figure 9 and Figure 10 show the reconstruction errors of ALADPL and GADPL of three test samples. From the figures, it is clearly seen that the reconstruction errors obtained by two methods are quite different. By selecting the local aspect sector of the inquiry sample adaptively, the differences between classes are improved, which benefits the classification rate.

To further show the superior performance of the proposed approach, the recognition rates of several recent SAR target recognition methods are also provided. Utilizing the same dataset, the sparse representation of the joint dynamic dictionary in [28] obtained a recognition rate of 96.48%; the two-dimensional slice Zernike moment sparse coding algorithm in [38] produced a recognition rate of 98.63%; the coupled dictionary learning approach in [39] gave a recognition rate of 96.07%. The best recognition rate of the proposed method reaches 99.60%, shown in Figure 7, which clearly demonstrated the advantage of the proposed method.

### 4.3. Classification Performance with Different Regularization Parameter Values

In the optimization process of the dictionary pair learning, the choice of regularization parameters *τ* and *λ* also has some impacts on the recognition performance of the algorithm. In order to show the effects of regularization parameters on the algorithm, *τ* is set to be a few typical values of 0.005, 0.01, 0.1, 0.5, 1, and *λ* set to 0.001, 0.005, 0.01, 0.05, 0.1, respectively. The aspect sector interval in ALADPL is set to 10° and *μ* is set to 0.0001. Those different regularization parameters are set in dictionary pair learning stage.

**Figure 11:**
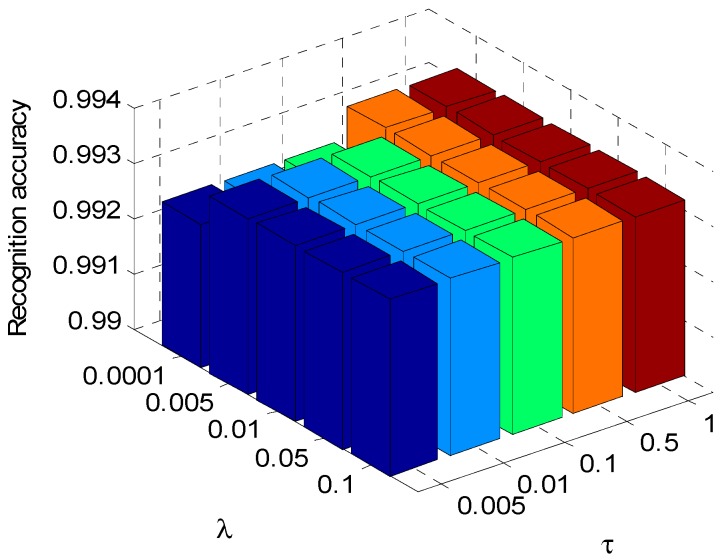
Recognition rate of ALADPL with the regularization parameters.

**Figure 12:**
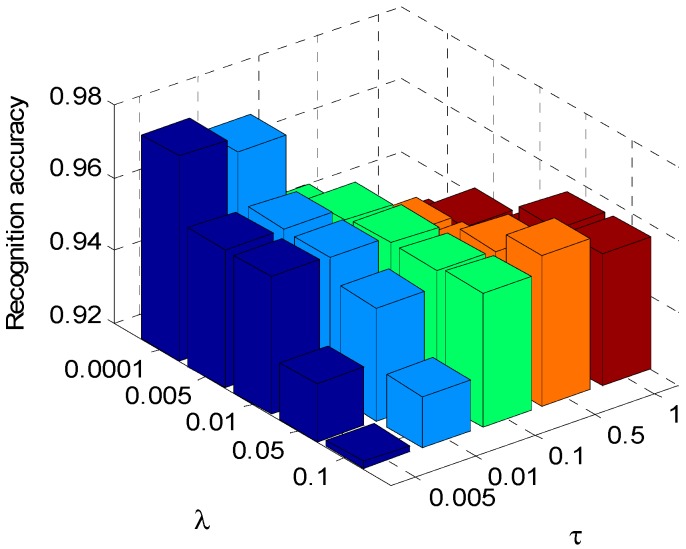
Recognition rate of GADPL with the regularization parameters.

Different regularization parameter values will product different dictionary pair, which we can observe the classification performance of the proposed approach. What’s more, the experiment with different regularization parameter values can offer a reference to apply this method to practical application. The results are respectively provided in Figure 11 and Figure 12.

It is seen that the correct classification rates of both ALADPL and GADPL exceed 90%, which illustrates excellent classification ability with the variations of regularization parameters. However, ALADPL is more robust than GADPL against the changes of regularization parameters, and the recognition performance of ALADPL only varies slightly.

### 4.4. Multiclass Targets Recognition

**Figure 13:**
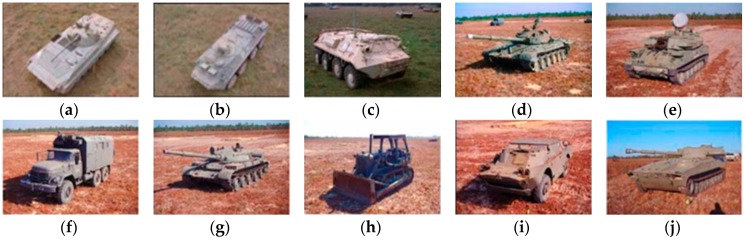
Optical images of ten targets. (**a**) BMP2; (**b**) BTR70; (**c**) BTR60; (**d**) T72; (**e**) ZSU; (**f**) ZIL; (**g**) T62; (**h**) D7; (**i**) BRDM2; (**j**) 2S1.

**Figure 14:**
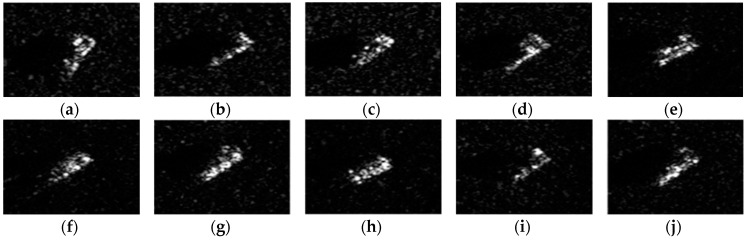
SAR image chips of three targets. (**a**) BMP2; (**b**) BTR70; (**c**) BTR60; (**d**) T72; (**e**) ZSU; (**f**) ZIL; (**g**) T62; (**h**) D7; (**i**) BRDM2; (**j**) 2S1.

In real scene, we could face with multiple targets rather than only three targets. For closer to practical application, in addition to the basic experiments under three classes, we also conducted experiments with ten-class targets to verify the classification performance of the proposed algorithm. The optical images of ten-class are shown in Figure 13, and their corresponding SAR images are shown in Figure 14. The numbers of training samples and testing samples are listed in Table 2. In this experiment, considering the high dimension of the data, we first extracted wavelet features to reduce dimensions and the aspect sector interval is set to 10°. Other regularization parameters are set as follow: set to 0.01, set to 0.01, set to 0.0001. Table 3 summarizes the classification performances of ALADPL, GADPL, sparse representation-based classification (SRC), collaborative representation-based classification (CRC) and LC-KSVD. The recognition rate of the proposed method is 93.07%, higher than other methods.

**Figure 15:**
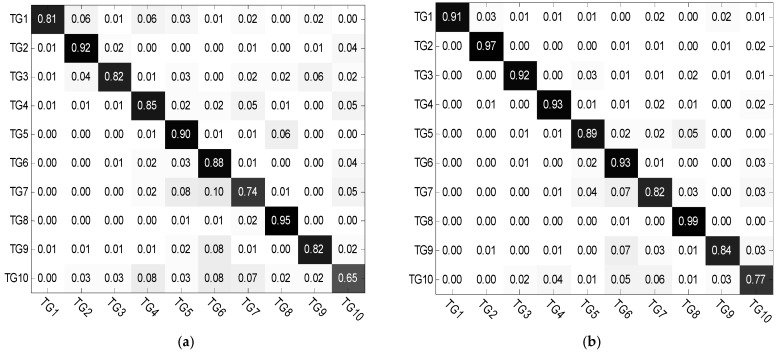

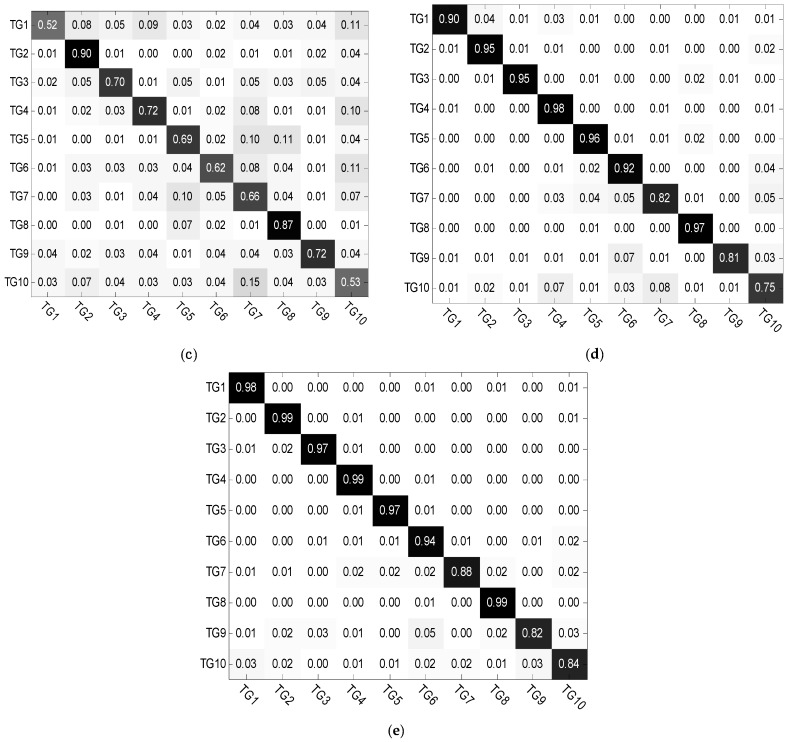
Confusion matrix of different methods. (**a**) SRC; (**b**) CRC; (**c**) LC-KSVD; (**d**) GADPL; (**e**) ALADPL.

We also list confusion matrices of the five methods respectively in Figure 15, in which the horizontal axis represents the real labels of the test samples and the vertical axis represents their predictive labels. From those confusion matrices, it is obvious that the correct recognition rates of TG7, TG9, and TG10 are lower than other targets. However, the conclusion is the same that the ALADPL has a better performance with multiclass targets for SAR target images classification. Under ten-class targets, we compare the performance of the proposed method with several recent SAR target recognition methods. The recognition rate of the method presented in [28] reached 91.48%. The method via supervised discriminative dictionary learning and sparse representation of the SAR-HOG feature in [29] obtained a recognition rate of 94.06%. The method based on information-decoupled representation proposed in [37] acquired a recognition rate of 94.88%. Our method achieves an equivalent performance with those state-of-the-art methods.

### 4.5. Robustness to Noise

**Figure 16:**
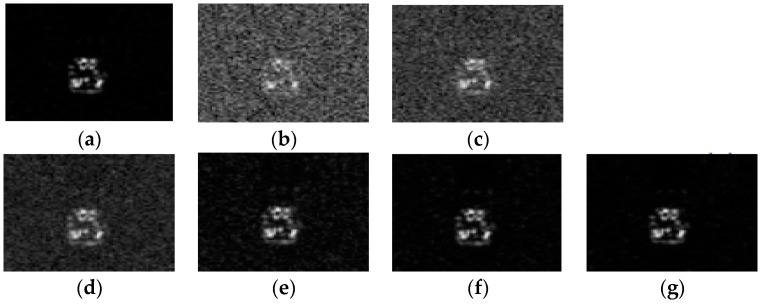
SAR images with different SNRs. (**a**) Original image; (**b**) 0 dB; (**c**) 10 dB; (**d**) 20 dB; (**e**) 30 dB; (**f**) 40 dB; (**g**) 50 dB.

**Figure 17:**
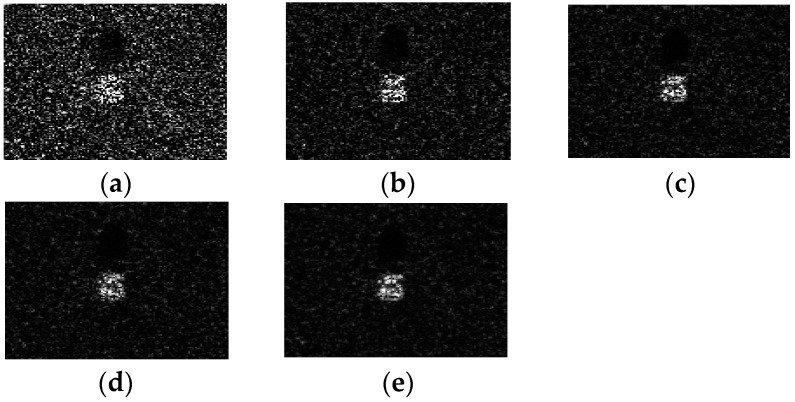
SAR images with different ENLs. (**a**) ENL = 0.5; (**b**) ENL = 0.6; (**c**) ENL = 1; (**d**) ENL = 1.5; (**e**) ENL = 2.

**Figure 18:**
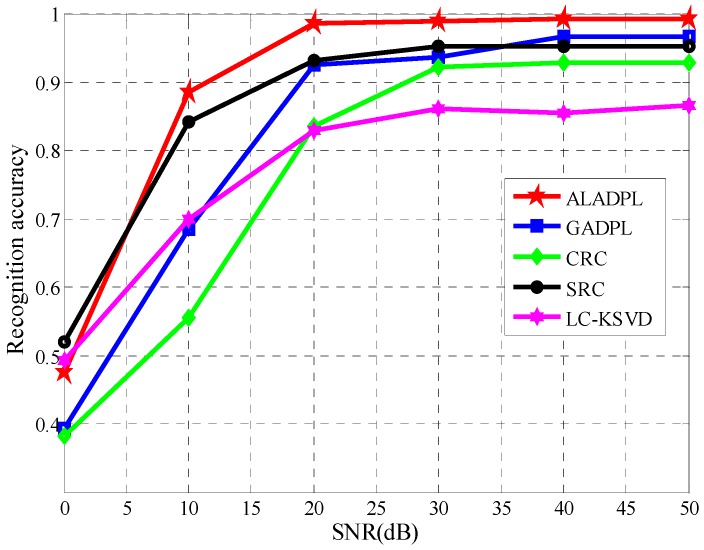
Performance of the algorithm with varying SNRs.

**Figure 19:**
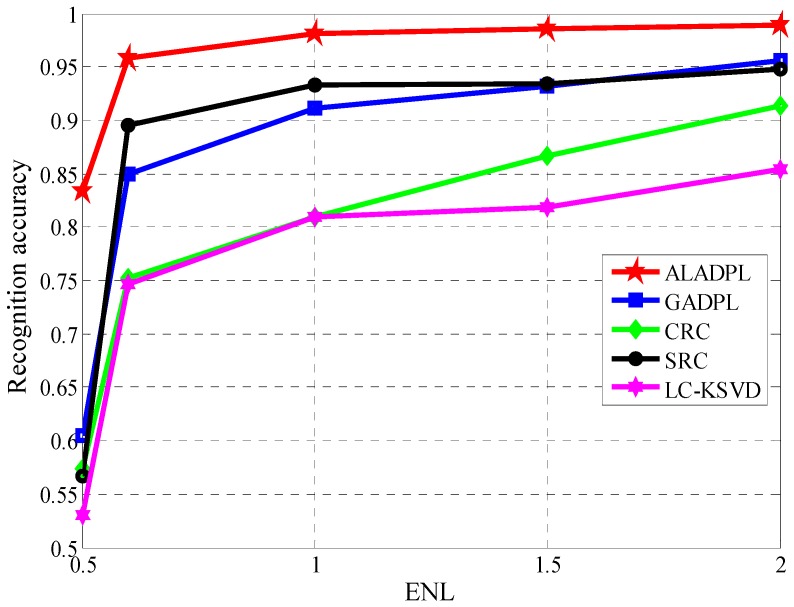
Performance of the algorithm with varying ENLs.

Due to the imaging mechanism and the influence of the electromagnetic environment, SAR target images are always contaminated by different noises such as the thermal noise and speckle noise. The noise reduces the quality of SAR images and increases the difficulty of correctly identifying the target. In order to show the anti-noise performance of ALADPL in practical applications, we added Gaussian noise with different signal to noise ratios (SNRs) and speckle noise with different equivalent numbers of looks (ENLs) to test samples in the experiments respectively. Figure 16 and Figure 17 show SAR images with two kinds of noise respectively. In this experiment, the aspect sector interval was still set to 10° and ALADPL is compared with several algorithms including GADPL, SRC, CRC, and LC-KSVD. The experimental results are provided in Figure 18 and Figure 19, Table 4 and Table 5. From Figure 18, it can be seen that with the increase of SNR, the recognition rates of the five methods are all improved, as expected. When the SNR is 50 dB, the ALADPL, GADPL, SRC, and LC-KSVD methods achieve the highest correct recognition rates of 99.26%, 96.71%, 95.29%, and 86.56%. Since SAR images are contaminated with severe noise at 0 dB, these methods obtained low classification accuracies. However, the classification accuracy of ALADPL is still more than GADPL about 8.2%, which means that the adaptive aspect sector selection is effective. When the SNR is 10 dB, ALADPL and SRC maintained the recognition rates of 88.61% and 84.18%, while the GADPL is only 68.54%. In addition, the ALADPL maintains a correct recognition rate over almost 90% in the SNR range from 10 dB to 50 dB. The anti-speckle noise performance is shown in Figure 19, and we can see that the proposed approach is obviously superior to other methods. Even when ENL is equal to 0.5, ALADPL can obtain the correct rate of 83.39% while other methods can only get the rates about 60%. When ENL is 1, ALADPL also maintain a high recognition rate of 98.07% compare with other methods, which demonstrates that ALADPL exhibits a good anti-speckle noise performance.

### 4.6. Experiments with Depression Angle Variations

**Figure 20:**
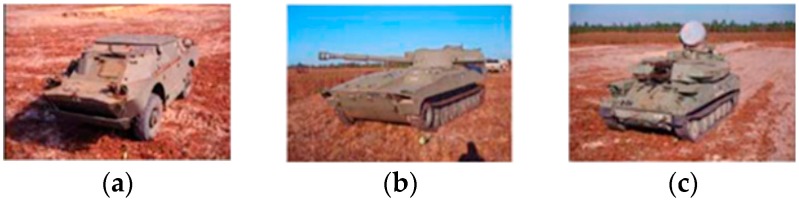
The optical images of three targets in large depression angle experiments. (**a**) BRDM2; (**b**) 2S1; (**c**) ZSU234.

**Figure 21:**
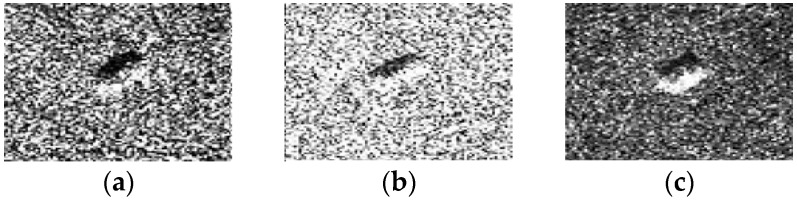
The microwave images of three targets in large depression angle experiments. (**a**) BRDM2; (**b**) 2S1; (**c**) ZSU234.

**Figure 22:**
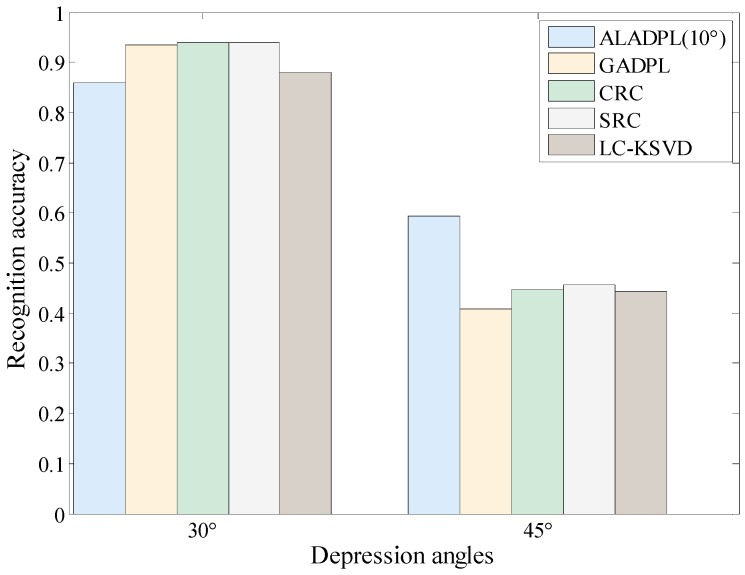
Performance of the algorithms in large depression angle experiment.

**Figure 23:**
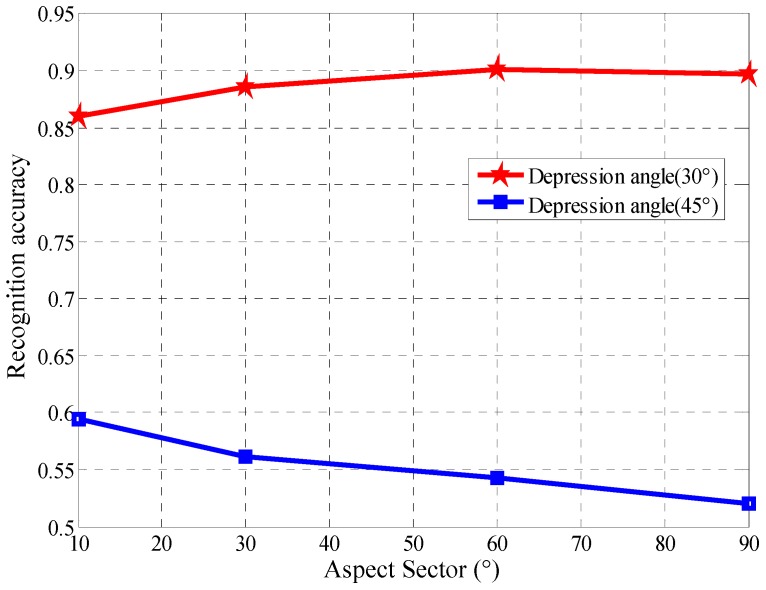
Performance of ALADPL with various aspect sector intervals.

In actual scenes, there is more likely to be a large difference between the depression angles of the test data set and training data set. In this experiment, ALADPL is evaluated with large depression angle variations. The dataset used in the experiment of depression angle variations is provided in Table 6. The three targets are BRDM2, 2S1, and ZSU234 shown in Figure 20 and Figure 21. The SAR images of the target at depression angle 17° are used for training, and images at depression angles of 30° and 45° are used for testing. The ALADPL aspect sector interval is set from 10° to 90°. The experimental results are depicted in Table 7. With the increased depression angle from 30° to 45°, the recognition rate of the five algorithms decreases significantly. When the depression angle is 30°, the correct recognition rates of ALADPL (10°) and LC-KSVD are only 86.0%, while the recognition rates of GADPL, SRC, and CRC methods are 93.40%, 93.75% and 93.87%. The reason behind of this is because SAR images are sensitive to changes of the depression angle. The difference in the depression angle between the training sample and the test sample produces a weak correlation between the training sample and the test sample. In fact, ALADPL is a local method. The mechanism behind local method is to make use of the strong correlation of local samples. Moreover, the confusion increases under weak correlation. The advantage of using a small amount of local aspect sector samples is lost. From Table 7 and Figure 22, when the depression angle between the training samples and the test samples further increases to 45°, the recognition rate of these methods decreases significantly, all less than 60%. In Figure 23, the performance of ALADPL with different depression angles against aspect sector intervals is provided. As expected, when the mismatch increases, the performance loss is also significant. Therefore, the robustness to the depression angle mismatch should be further studied.

## 5. Conclusions

This paper discusses SAR target image classification based on adaptive local aspect dictionary pair learning and evaluates the performance of the proposed algorithm with the MSTAR database. The experimental results could offer a reference to apply the method in practical application, such as how to choose suitable local aspect sector interval and regularization parameters. In the proposed method, the local aspect sector is selected based on regularized non-negative sparse learning, and the aspect information of the training samples is reasonably explored. Experimental results also confirm that the proposed method presents a comparative recognition performance with state-of-art methods for SAR image target recognition. Compared with the dictionary learning using global aspect training dataset, it is more robust to the noise and the variation of regularization parameters. Compared with other SAR target images classification algorithms, the proposed method provides the following advantages. First, the method exploits the local aspect characteristics of test samples for SAR image targets classification. Second, the dictionary pair learned from local aspects is more compact and has a strong inter-class discrimination power. Moreover, the number of training samples located in a local aspect is less than the global aspects, which saves the computing costs during dictionary learning. Although we perform quite deeply experimental analysis, we lack of complete scenes and other structures or weapons of SAR images dataset to evaluate the proposed method. In future research, we will try our best to collect other SAR images dataset to improve our research.

## Figures and Tables

**Table 1:** The types and numbers of training and testing data sets.

Target	1	2	3
	BMP2	BTR70	T72
Training (17°)	233 (sn-9563)	233 (sn-c71)	232 (sn-132)
	232 (sn-9566)		231 (sn-812)
	233 (sn-c21)		228 (sn-s7)
Testing (15°)	195 (sn-9563)	196 (sn-c71)	196 (sn-132)
	196 (sn-9566)		195 ( sn-812)
	196 (sn-c21)		191 (sn-s7)

**Table 2:** The dataset used in multiclass experiments.

Target	TG1	TG2	TG3	TG4	TG5	TG6	TG7	TG8	TG9	TG10
	BMP2	BTR70	BTR60	T72	ZSU	ZIL	T62	D7	BRDM2	2S1
Training (17°)	233 (sn-9563)	233	256	232 (sn-c21)	299	299	299	299	298	299
Testing (15°)	195 (sn-9563)	196	195	196 (sn-c21)	274	274	273	274	274	274

**Table 3:** The recognition rate obtained in multiclass experiments.

Method	SRC	CRC	LC-KSVD	GADPL	ALADPL
**Recognition rate**	0.8318	0.8920	0.6911	0.8961	0.9307

**Table 4:** Performance of the algorithm with different SNRs.

Method	SNR (dB)					
	0	10	20	30	40	50
**ALADPL**	47.56%	88.61%	98.64%	98.98%	99.21%	99.26%
**GADPL**	39.34%	68.54%	92.63%	96.32%	96.66%	96.71%
**CRC**	38.21%	55.56%	83.62%	92.29%	92.86%	92.80%
**SRC**	54.88%	84.63%	0.9314%	95.29%	95.24%	95.29%
**LC-KSVD**	49.32%	66.38%	83.01%	86.13%	85.48%	86.56%

**Table 5:** Performance of the algorithm with different ENLs.

Method	ENL				
	0.5	0.6	1	1.5	2
**ALADPL**	83.39%	95.80%	98.07%	98.53%	98.92%
**GADPL**	60.49%	84.98%	91.10%	93.20%	95.58%
**CRC**	57.43%	75.23%	80.95%	86.62%	91.33%
**SRC**	56.75%	89.51%	93.25%	93.42%	94.84%
**LC-KSVD**	53.06%	74.60%	80.95%	81.92%	85.37%

**Table 6:** Dataset used in large depression angle experiment.

Target	1	2	3
	BRDM2	2S1	ZSU234
Training Set (17°)	298	299	299
Testing Set (30°)	287	288	288
Testing Set (45°)	303	303	303

**Table 7:** Performance of the algorithms in large depression angle experiments.

Depression	Method							
	SRC	CRC	LC-KSVD	GADPL	ALADPL (10°)	ALADPL (30°)	ALADPL (60°)	ALADPL (90°)
30°	0.9375	0.9387	0.8808	0.9340	0.8600	0.8854	0.9016	0.8970
45°	0.4576	0.4466	0.4433	0.4081	0.5941	0.5611	0.5424	0.5204
